# Comparison of Computed and Acquired DWI in the Assessment of Rectal Cancer: Image Quality and Preoperative Staging

**DOI:** 10.3389/fonc.2022.788731

**Published:** 2022-03-18

**Authors:** Yihan Xia, Lan Wang, Zhiyuan Wu, Jingwen Tan, Meng Fu, Caixia Fu, Zilai Pan, Lan Zhu, Fuhua Yan, Hailin Shen, Qianchen Ma, Gang Cai

**Affiliations:** ^1^ Department of Radiology, Ruijin Hospital, Shanghai Jiao Tong University of Medicine, Shanghai, China; ^2^ Department of MR Application Development, Siemens Shenzhen Magnetic Resonance Ltd, Shenzhen, China; ^3^ Department of Radiology, Suzhou Kowloon Hospital, Shanghai Jiao Tong University of Medicine, Suzhou, China; ^4^ Department of Pathology, Ruijin Hospital, Shanghai Jiao Tong University of Medicine, Shanghai, China; ^5^ Department of Radiation Oncology, Ruijin Hospital, Shanghai Jiao Tong University of Medicine, Shanghai, China

**Keywords:** rectal cancer, diffusion-weighted imaging, computed diffusion images, image quality, diagnostic performance

## Abstract

**Objective:**

The aim of the study was to evaluate the computed diffusion-weighted images (DWI) in image quality and diagnostic performance of rectal cancer by comparing with the acquired DWI.

**Methods:**

A total of 103 consecutive patients with primary rectal cancer were enrolled in this study. All patients underwent two DWI sequences, namely, conventional acquisition with b = 0 and 1,000 s/mm^2^ (aDWI_b1,000_) and another with b = 0 and 700 s/mm^2^ on a 3.0T MR scanner (MAGNETOM Prisma; Siemens Healthcare, Germany). The images (b = 0 and 700 s/mm^2^) were used to compute the diffusion images with b value of 1,000 s/mm^2^ (cDWI_b1,000_). Qualitative and quantitative analysis of both computed and acquired DWI images was performed, namely, signal-to-noise ratio (SNR), contrast-to-noise ratio (CNR), and signal intensity ratio (SIR), and also diagnostic staging performance. Interclass correlation coefficients, weighted κ coefficient, Friedman test, Wilcoxon paired test, and McNemar or Fisher test were used for repeatability and comparison assessment.

**Results:**

Compared with the aDWI_b1,000_ images, the cDWI_b1,000_ ones exhibited significant higher scores of subjective image quality (all P <0.050). SNR, SIR, and CNR of the cDWI_b1,000_ images were superior to those of the aDWI_b1,000_ ones (P <0.001). The overall diagnostic accuracy of computed images was higher than that of the aDWI_b1,000_ images in T stage (P <0.001), with markedly better sensitivity and specificity in distinguishing T1–2 tumors from the T3–4 ones (P <0.050).

**Conclusion:**

cDWI_b1,000_ images from lower b values might be a useful alternative option and comparable to the acquired DWI, providing better image quality and diagnostic performance in preoperative rectal cancer staging.

## Introduction

The incidence and mortality of colorectal cancer (CRC) in China has been increasing yearly, 30–35% of which are rectal cancers (1). Patients with rectal cancers are treated with surgery only or a combination of surgery and chemoradiation therapy (CRT)according to their preoperative staging (2). Therefore, accurate preoperative staging is critical for decision-making in clinical practice.

During the past decade, MRI has been proven to be the most accurate staging modality in patients with rectal cancer (3). Diffusion-weighted imaging (DWI) with two b-values has been validated in detecting and staging primary rectal cancer and also restaging and predicting tumor response after neoadjuvant CRT (4). However, appropriate b-value of DWI is of significance in achieving balance among image quality, diagnostic capability, and acquisition time. On one hand, T2 ‘shine-through effects’ of lower-b value (<800 s/mm^2^) images often reduce the tumor conspicuity and hamper the detection and staging/restaging of rectal cancer (5–8). On the other hand, higher b-value DWI images of the rectum always suffer from low image quality, namely, relatively low SNR in the high-b-value images or distortion artifact due to gas within the rectum (9, 10). To improve the SNR of the high-b-value images, normally large number of averages is used, which would prolong the scanning time and thus increases the motion sensitivity during the whole acquisition. Moreover, high-b images are more prone to being contaminated by the distortion artifact due to the applied high gradient amplitude. To our knowledge, DWI with a b-value of greater than 800 s/mm^2^ specially 1,000 s/mm^2^ has been accepted as the standard high b-value DWI in rectal cancer imaging achieving the balance in clinical practice (11–15). Computed DWI is a voxel-wise apparent diffusion efficient (ADC)-based postprocess method to calculate random high b-value images from DWI images acquired with at least two different lower b-values (9, 15). Studies have reported the usefulness of this technique for the detection of hepatic metastases, pancreatic adenocarcinoma, and grading prostate cancer (9, 16–18). Based on previous studies, due to computed DWI images from lower b-values with less acquisition time, poor SNR, and image distortion related to direct high b-value measurements may be avoided while adequate cancer detection rate maintained. However, the efficacy of computed high b-value DWI in rectal cancer imaging has not been determined.

Therefore, the purpose of our study aimed to evaluate the value of the computed DWI by assessing its image quality and diagnostic performance on rectal cancer, comparing with the conventionally acquired DWI.

## Material and Methods

### Patients

**Figure 1:**
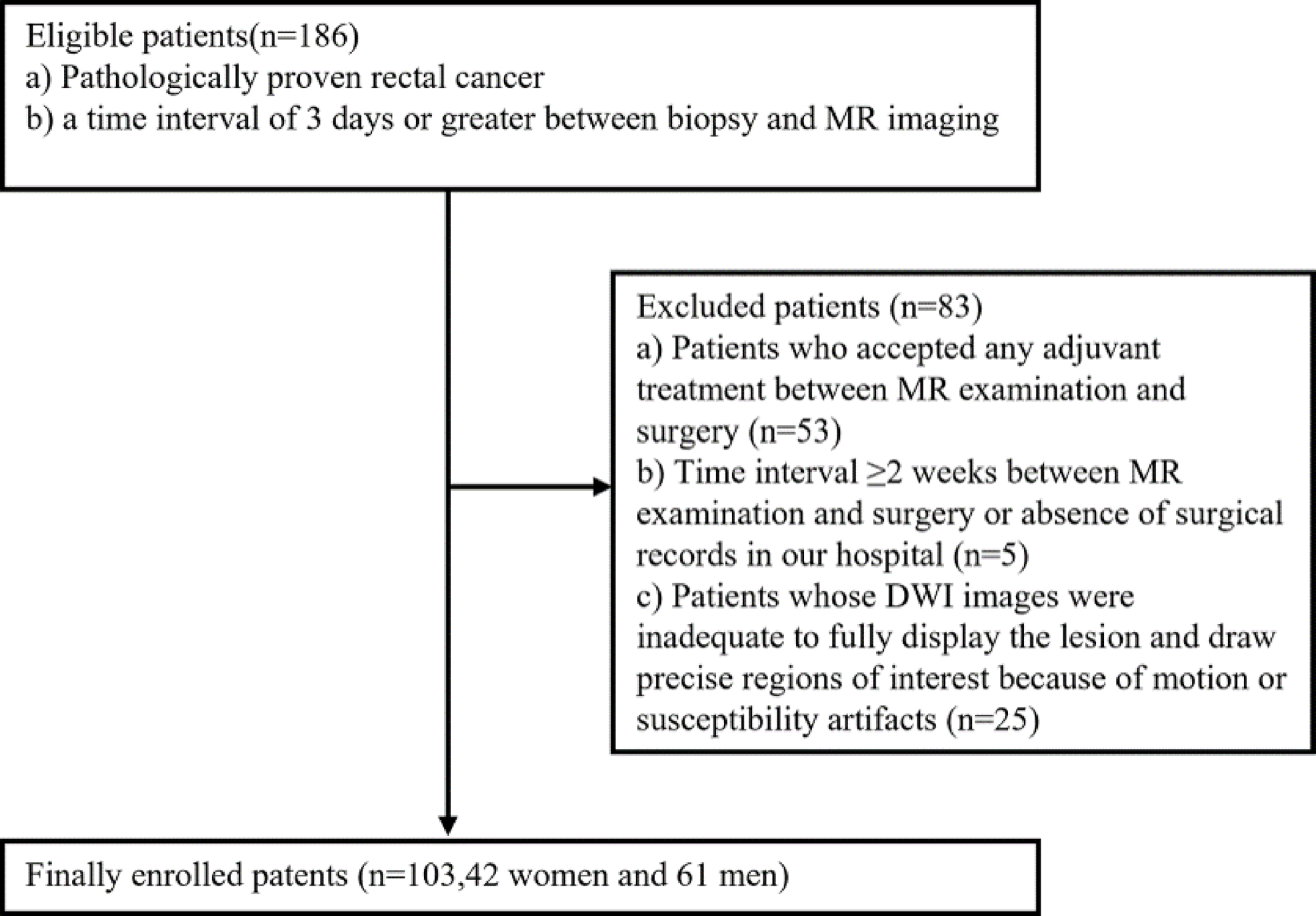
Flowchart of the study.

This retrospective study was approved by the Institutional Review Board and the requirement for formal informed consent from all patients was waived. Between February 2020 and March 2021, patients whose endoscopic biopsy results had proven or raised suspicions of primary rectal adenocarcinoma were referred for a rectal MR examination. A total of 186 patients who satisfied the following criteria were initially enrolled in this study: pathologically proven rectal adenocarcinomas with surgical specimens and a time interval of 3 days or greater between biopsy and MR imaging. The following exclusion criteria were employed for further exploration: a) Patients who accepted any adjuvant treatment between MR examination and surgery (n = 53); b) Time interval ≥2 weeks between MR examination and surgery or absence of surgical records in our hospital (n = 5); c) Patients whose DWI images were inadequate to fully display the lesion and draw precise regions of interest because of motion or susceptibility artifacts (n = 25). Finally, 103 patients (median age, 64 years; range, 30–91 years), consisting of 42 women and 61 men, were finally included in our study (**Figure 1**). Among the final population, 58 patients were staged as T3–4/N1–2M0 in preoperative radiological reports, yet they still underwent radical surgery taking multi-disciplinary team discussion and desire into consideration of the patients.

### MR Image Acquisition

All MRI examinations were performed on a MAGNETOM Prisma 3.0 T MRI scanner (Siemens Healthcare, Erlangen, Germany) with an 18-channel body coil. Before the MR scanning, each subject fasted for 4 h, and a 40-ml enema (Glycerol Enema, Shanghai, China) was rectally administered 1–2 h to achieve better contrast between the tumor and the rectal lumen for detecting small lesions (19). All examinations followed a standard procedure in our hospital. First, a sagittal T2-weighted turbo spin-echo sequence without fat saturation was obtained for the selection of both oblique axial and coronal images, which were orthogonal and parallel to the long axis of the tumor, respectively. Two DWI sequences, one with b-values of 0 and 1,000 s/mm^2^ (aDWI_b1,000_) and another with b-values of 0 and 700 s/mm^2^ were performed in the oblique axial plane by using a single-shot echo-planar imaging sequence with comparable parameters (**Table 1**). The diffusion gradients were set along three orthogonal directions. A dynamic field correction is applied inline to correct eddy current-induced geometric distortion for all diffusion-weighted images.

**Table 1:** Imaging Protocol Parameters for DWI and HRT2WI Sequences.

	HR T2WI	aDWI_b1,000_	DWI*
Sequence	Turbo spin echo	Single-shot echo-planar	Single-shot echo-planar
TR/TE (ms)	9,010/108	4,780/89	4,780/91
No. of slices	24	24	24
Thickness (mm)	3	3	3
Gap (mm)	0.3	0.3	0.3
Bandwidth (Hz/pixel)	381	1,116	1,116
Field of View (mm^2^)	20 × 20	26 × 26	26 × 26
Voxel size (mm^3^)	0.3 × 0.3 × 3.0	1.9 × 1.9 × 3.0	1.9 × 1.9 × 3.0
Time (min’ s’’)	2’15’’	2’28’’	2’28’’
b-values (s/mm^2^)	/	0, 1,000	0, 700
No. of signal averages**	2	1, 2	1, 2

HR T2WI, high-resolution T2-weighted imaging; aDWI_b1,000_, acquired diffusion-weighted imaging with b-value of 0 and 1,000 s/mm^2^; TR, repetition time; TE, echo time.

*DWI with b-value of 0 and 700 s/mm^2^.

**Number of averages is in sequence corresponding to b-values above.

### Imaging Analysis

#### Computed Diffusion Images

The DWI images with b = 0 and 700 s/mm^2^ were imported into a prototype Diffusion Toolbox in syngo.*via* Frontier (Siemens Healthcare, Germany) to compute the diffusion images with b-value of 1,000 s/mm^2^ (cDWI_b1,000_) using a mono-exponential diffusion decay model.

The cDWI_b1,000_ and aDWI_b1,000_ diffusion images were copied and numbered randomly with corresponding T2-weighted (T2W) images by LZ, who was the only one who knew the numbering results.

#### Subjective Assessment

Interpretation of rectal MRI images was independently performed by two radiologists with 7 years (YX) and 10 years (LW) of abdominal imaging experience, respectively. They were both blinded to the corresponding patient clinical information and which diffusion images they assessed. Each radiologist evaluated diffusion images of 40–48 patients once every two weeks with corresponding T2W images as reference (20–25). The two radiologists worked separately. The assessments included image quality, tumor location and TN stages. The author (LZ) recorded and sorted all results according to the number of diffusion images. If the results differed when using the same diffusion images, a senior imaging expert (ZP), who had 25 years of experience in interpretation images of rectal masses was consulted to achieve consensus.

For subjective assessment of image quality, the two radiologists scored the diffusion images. The evaluation of image quality was analyzed using a Likert scale with scores ranging from 1 to 4 (26):

1. Sharpness (1 = not sharp; 2 = a little sharp; 3 = moderately sharp; 4 = sharp);

2. Distortion (1 = severe; 2 = moderate; 3 = mild; 4 = no distortion);

3. Artifacts (1 = serious, difficult to diagnose; 2 = moderate; 3 = mild; 4 = absence of artifacts);

4. Lesion conspicuity (1 = difficult to find; 2 = minimally perceivable; 3 = recognizable; 4 = easy to detect, good contrast of lesion and background noise).

Total subjective image quality scores were then obtained by adding the four values above.

#### Assessment of Local Preoperative TN Staging and Tumor Location

The two radiologists evaluated the given cases on the postprocessing workstation (syngo.*via*, Siemens Healthcare). The criteria used for determining the TN stage were based on the eighth edition of American Joint Committee on Cancer Staging Manual for rectal cancer (27). Carcinoma growing within submucosa layer was staged as T1, while T2 tumor extended submucosa layer and penetrates into but within muscularis propria. Carcinoma of T3 stage extended into subserosa and/or perirectal tissue, while tumor invaded the surface of the visceral peritoneum or other organs/structures staged as T4. For N staging, N0 represents no regional lymph node metastasis; N1 for metastases of 1 to 3 regional lymph nodes occurred, and N2 means metastases of no less than 4 regional lymph nodes occurred. Morphological criteria for suspicion of positive lymph node included a short-axis diameter of ≥5 mm, an irregular border, mixed-signal intensity or a round shape, and diffusion criteria (high signal intensity on images of high b-values) (28, 29).

**Figure 2:**
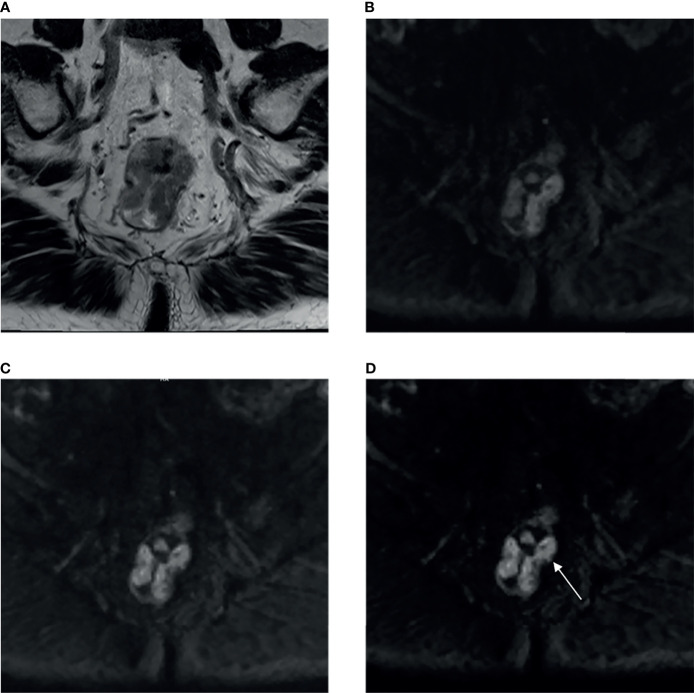
A 85-year-old male patient with moderately-to-poorly differentiated rectal adenocarcinoma (T3 stage). **(A)** High-resolution T2WI exhibited irregular thickening of rectal wall. The aDWI_b1,000_ **(B)**, aDWI_b700_ **(C)** and cDWI_b1,000_ **(D)** images showed corresponding rectal wall with partially high signal intensity. When using T2WI and aDWI_b1,000_ images, two radiologists considered the tumor invades but does not penetrate muscularis propria as T2 stage, while combining T2WI and cDWI_b1,000_ images, the papillary (arrow) suggested tumor invades subserosa through muscularis propria, both radiologists considered the tumor as T3 stage. aDWI_b1,000_, acquired diffusion-weighted imaging with b-value of 0 and 1,000 s/mm^2^; aDWI_b700_, acquired diffusion-weighted imaging with b-value of 0 and 700 s/mm^2^; cDWI_b1,000_, computed diffusion images with b-value of 1,000 s/mm^2^ from 0 and 700 s/mm^2^.

The tumor location of all cases was grouped according to the distances between the lowest part of the tumor and anal verge on sagittal T2WI images. The lower/distal rectal cancer was defined when the distance was less than 5 cm, while mid-rectal cancer was defined when the distance ranged from 5 to 10 cm. The others (10–15 cm from the anal verge) were grouped as upper rectal cancers (**Figure 2**).

#### Objective Assessment of Image Quality

The signal-to-noise ratio (SNR) was defined as the ratio between the mean signal intensity (SI) inside the tumor (*S_tumor_*) and the standard deviation (SD) of background noise (*SD_background_*) using the following equation:


$$\text{SNR}=S_{tumor}/SD_{background}$$

The contrast-to-noise ratio (CNR) was defined as the ratio of the mean signal intensity difference between tumor (*S_tumor_*) and normal tissue (*S_tissue_*) divided by the standard deviation of the tumor (*SD_tumor_*) and normal tissue (*SD_tissue_*) using the following equation: $CNR=\left|S_{tumor}-S_{tissue}\right|/\sqrt{SD_{tumor}{}^{2}+SD_{tissue}{}^{2}}$ . The signal-intensity-ratio (SIR) was defined as the ratio of the mean signal intensity difference between tumor (*S_tumor_*) and normal tissue (*S_tissue_*) using the following equation: $\text{SIR}=S_{tumor}/S_{tissue}$ .

**Figure 3:**
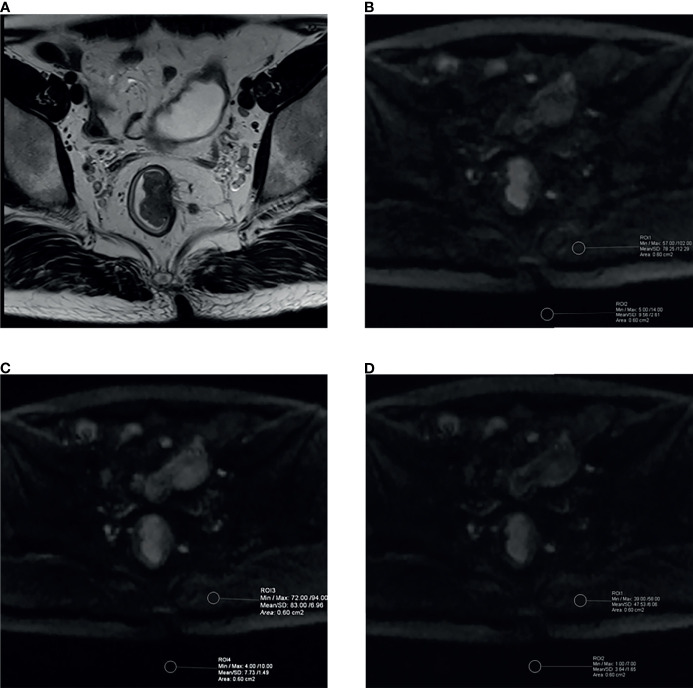
Example images for delineating ROI and comparison between aDWI_b1,000_ and cDWI_b1,000_ images in a 39-year-old male patient with moderately differentiated rectal adenocarcinoma (T2 stage). **(A)** High-resolution T2WI exhibited irregular thickening of rectal wall. The aDWI_b1,000_ **(B)**, aDWI_b700_ **(C)** and cDWI_b1,000_ **(D)** images showed corresponding rectal wall with partially high signal intensity. The two circle ROIs were distributed in the background and gluteus maximus on the same slices and the same locations on both diffusion images to compute objective parameters. SNR, CNR and SIR were 147.67 vs. 257.90, 8.68 vs. 13.88 and 5.17 vs. 5.23 for aDWI_b1,000_ and cDWI_b1,000_ images, respectively. The cDWI_b1,000_ images achieved higher scores than aDWI_b1,000_ images by both readers in terms of subjective image parameters. aDWI_b1,000_, acquired diffusion-weighted imaging with b-value of 0 and 1,000 s/mm^2^; aDWI_b700_ = acquired diffusion-weighted imaging with b-value of 0 and 700 s/mm^2^; cDWI_b1,000_ = computed diffusion images with b-value of 1,000 s/mm^2^ from 0 and 700 s/mm^2^.

ROIs in this study were drawn by the two radiologists mentioned above using the single-section method (30) and the principle as follows. For lesions, a single freehand ROI was defined by tracing a line around the perceived tumor margins on the section containing the largest tumor area, the mean signal intensity and its standard deviation were obtained as *S_tumor_* and *SD_tumor_*. Similarly, a single circle ROI was drawn in homogeneous normal tissue, which was located on the same slice but far from the tumor area, and then the mean signal intensity and standard deviation were recorded as *S_tissue_* and *SD_tissue_*.The tissues we included to compute CNR or SIR as follows: the mesorectum (CNR/SIR-mesorectum), gluteus maximus (CNR/SIR-gluteus), subcutaneous fat (CNR/SIR-fat), and the interface between gluteus maximus and subcutaneous fat (CNR/SIR-interface) to conduct a comprehensive assessment. Besides, a single circle ROI was placed in the signal-free background as mentioned above (**Figure 3**), and its standard deviation was recorded as *SD_background_*. The above quantitative assessment was performed on a commercial postprocessing workstation (syngo.*via*, Siemens Healthcare).

### Statistical Analysis

All parameters were first tested by using the Kolmogorov–Smirnov test for normality analysis. The comparison of subjective image quality analysis scores was performed using the Friedman test. Differences in objective image quality parameters (SNR, CNR, and SIR) between cDWI_b1,000_ and the aDWI_b1,000_ images were compared using the paired Wilcoxon test. The McNemar or Fisher exact probability test was performed to evaluate the difference (namely, accuracy, sensitivity, and specificity) of TN staging between both techniques. The interobserver variability for subjective image quality parameters, diagnostic performance, and consistency between preoperative MR and histopathologic TN staging was evaluated using weighted kappa statistics. The interobserver variability for objective image quality parameters was assessed using the interclass correlation coefficient (ICC) test. The ICC and κ value obeyed the grouping criteria as follows: poor agreement: 0.00–0.20; fair agreement: 0.21–0.40; moderate agreement: 0.41–0.60; good agreement: 0.61–0.80; and excellent agreement: 0.81–1.00.

Statistical analysis was performed by using statistical software (SPSS 26.0, SPSS, IBM; and Medcalc17.9, Medcalc, Mariakerke, Belgium). Results with P <0.050 were considered statistically significant.

## Results

### Patients and Pathologic Results

A total of 103 patients (61 men and 42 women) with a mean age of 63.58 ± 10.81 years and a range of 30–91 years were included in the final analysis. A total of 55 patients had mid-rectal cancer, while 32 and 16 patients had lower and upper rectal cancers, respectively. All pathologic TN staging (pTN) was conducted after surgery. Among these, 12 (11.65%) were staged as pT1, 37 (35.92%) as pT2, 49 (47.57%) as pT3 and 5 (4.85%) as pT4. As for histopathologic N staging, 65 (63.11%) were staged as pN0, 28 (27.18%) as pN1, and 10 (9.71%) as pN2.

### Assessment of Local Preoperative TN Staging

#### T Staging of Rectal Cancer

The accuracy, sensitivity, and specificity of each T stage are shown below (**Table 2**). Compared to T2W and aDWI_b1,000_ images, evaluation with T2W and cDWI_b1,000_ images demonstrated higher overall MR T staging accuracy and excellent consistency with pathologic T staging (accuracy: 93.20% vs. 69.90%; κ value: 0.892 vs. 0.530, respectively, all P <0.001).

**Table 2:** T staging of rectal cancer using cDWI_b1,000_ and aDWI_b1,000_ images.

Pathologic T stages (n)	cDWI_b1,000_				aDWI_b1,000_			
	T1	T2	T3	T4	T1	T2	T3	T4
pT1 (12)	10	2	0	0	5	7	0	0
pT2 (37)	0	35	2	0	3	25	9	0
pT3 (49)	0	1	46	2	0	6	37	6
pT4 (5)	0	0	0	5	0	0	0	5
Accuracy (%)	98.06	95.15	95.15	98.06	90.29	75.73	79.61	94.17
Sensitivity (%)	83.33	94.60	93.88	100	41.67	67.57	75.51	100
Specificity (%)	100	95.45	96.30	97.96	96.70	80.30	83.33	93.88

Total accuracy rate = 93.20% for calculated sequence, κ = 0.892, P <0.001.

Total accuracy rate = 69.90% for DWI sequence, κ = 0.530, P <0.001.

pTx, pathologic T staging; aDWI_b1,000_, acquired diffusion-weighted imaging with b-value of 0 and 1,000 sec/mm^2^; cDWI_b1,000_, computed diffusion images with b-value of 1,000 s/mm^2^ from 0 and 700 s/mm^2^.

In detail, there were no significant differences in accuracy, sensitivity, and specificity of both cDWI_b1,000_ and aDWI_b1,000_ images to diagnostic T4 tumors (all P >0.050). The cDWI_b1,000_ images exhibited higher accuracy in determining T1, T2, and T3 rectal cancers (T1: χ^2^ = 5.640, P = 0.018; T2: χ^2^ = 15.537, P <0.001; T3: χ^2^ = 11.223, P <0.001), and so as the sensitivity (T1: χ^2^ = 37.951; T2: χ^2^ = 24.414; T3: χ^2^ = 13.342, all P <0.001). In combination with T2WI, the cDWI_b1,000_ and aDWI_b1,000_ images had similar diagnostic specificity in staging T1 cancers (χ^2^ = 3.439, P = 0.064), while the former one performed better in identifying T2 and T3 neoplasms (T2: χ^2^ = 11.040, P <0.001; T3: χ2 = 9.425, P = 0.002).

Speaking of distinguishing T1–2 from T3–4 rectal cancers, cDWI_b1,000_ images combining T2W images demonstrated higher both sensitivity (95.92% vs. 81.63% for cDWI_b1,000_ and aDWI_b1,000_, respectively; χ^2^ = 10.502, P = 0.001) and specificity (98.15% vs. 88.89%; χ^2^ = 7.252, P = 0.007, respectively).

### N Staging of Rectal Cancer

The accuracy, sensitivity, and specificity of each N stage are exhibited below (**Table 3**). With T2W and aDWI_b1,000_ images, the overall MR accuracy was 68.93%, and the κ value was 0.460 (P <0.001). While evaluated with T2W and cDWI_b1,000_ images, the overall MR accuracy was improved to 78.64%, and the κ value was increased to 0.616 (P <0.001).

**Table 3:** N staging of rectal cancer using cDWI_b1,000_ and aDWI_b1,000_ images.

Pathologic N stages (n)	cDWI_b1,000_			aDWI_b1,000_		
	N0	N1	N2	N0	N1	N2
pN0 (65)	50	13	2	45	17	3
pN1 (28)	4	24	0	5	19	4
pN2 (10)	0	3	7	0	3	7
Accuracy (%)	81.55	80.58	95.15	75.73	71.84	90.29
Sensitivity (%)	76.92	85.71	70.00	69.23	67.86	70.00
Specificity (%)	89.47	78.67	97.85	86.84	73.33	92.47

Total accuracy rate = 78.64% for calculated sequence, κ = 0.616, P <0.001.

Total accuracy rate = 68.93% for DWI sequence, κ = 0.460, P <0.001.

pNx, pathologic N staging; aDWI_b1,000_ = acquired diffusion-weighted imaging with b-value of 0 and 1,000 s/mm^2^; cDWI_b1,000_ = computed diffusion images with b-value of 1,000 s/mm^2^ from 0 and 700 s/mm^2^.

No significantly different diagnostic accuracy, sensitivity, and specificity of the cDWI_b1,000_ and aDWI_b1,000_ images were found in the N staging except that the latter ones suffered from lower sensitivity in determining N1 tumors (85.71% vs. 67.86% for cDWI_b1,000_ and aDWI_b1,000_, respectively; χ^2^ = 9.161, P = 0.003).

In terms of discerning patients without metastatic nodes, the cDWI_b1,000_ and aDWI_b1,000_ images showed similarly moderate sensitivity (76.92% vs. 69.23% for cDWI_b1,000_ and aDWI_b1,000_, respectively; χ^2^ = 10.502, P = 0. 215) and high specificity (89.47% vs. 86.84%; χ^2^ = 0.339, P = 0.560).

### Subjective Assessment of Image Quality

The comparison of subjective image quality parameters between cDWI_b1,000_ and aDWI_b1,000_ images based on the 4-point scoring system is presented in **Table 4**. The image quality of cDWI_b1,000_ was superior to that of aDWI_b1,000_ for all criteria, namely, sharpness (4.330 ± 0.493 vs. 3.505 ± 0.655, P <0.001), distortion (4.165 ± 0.643 vs. 4.097 ± 0.748, P = 0.008), artifacts (4.262 ± 0.593 vs. 4.194 ± 0.611, P = 0.035), lesion conspicuity (4.252 ± 0.589 vs. 4.029 ± 0.649, P <0.001) and total subjective imaging quality score (17.010 ± 2.089 vs. 15.825 ± 2.198, P <0.001).

**Table 4:** Comparisons of Image Quality Between cDWI_b1,000_ and aDWI_b1,000_ images.

Parameters	cDWI_b1,000_	aDWI_b1,000_	P-value
Sharpness*	4.330/171.471 ± 0.493	3.505/135.529 ± 0.655	0.000
Distortion*	4.165/104.903 ± 0.643	4.097/102.097 ± 0.748	0.008
Artifacts*	4.262/106.175 ± 0.593	4.194/100.825 ± 0.611	0.035
Lesion conspicuity*	4.252/112.422 ± 0.589	4.029/94.578 ± 0.649	0.000
Total subjective image quality*	17.010/121.126 ± 2.089	15.825/85.874 ± 2.198	0.000
SNR	273.649 ± 56.100	190.923 ± 41.786	0.000
CNR (mesorectum)	12.043 ± 2.665	10.702 ± 2.146	0.000
CNR (gluteus)	10.694 ± 2.846	9.625 ± 2.203	0.000
CNR (fat)	9.063 ± 2.572	7.801 ± 2.134	0.000
CNR (interface)	13.977 ± 3.422	12.675 ± 2.557	0.000
SIR (mesorectum)	8.099 ± 3.203	6.652 ± 1.862	0.000
SIR (gluteus)	4.804 ± 1.268	4.505 ± 1.214	0.000
SIR (fat)	3.403 ± 0.939	3.018 ± 0.892	0.000
SIR (interface)	17.850 ± 5.451	15.052 ± 3.325	0.000

Data are means and standard deviations (averages between readers).

*Data are means/mean rank and standard deviations (averages between readers).

SNR, signal-to-noise ratio; CNR, contrast-to-noise ratio; SIR, signal intensity ratio;

aDWI_b1,000_, acquired diffusion-weighted imaging with b-value of 0 and 1,000 s/mm^2^; cDWI_b1,000_, computed diffusion images with b-value of 1,000 s/mm^2^, from 0 and 700 s/mm^2^.

### Objective Assessment of Image Quality

The comparison of objective image quality parameters between both methods is shown in **Table 4**. The overall SNR of cDWI_b1,000_ was significantly higher than those of aDWIb_1,000_ (P <0.001). It was remarkable that CNR and SIR of the mesorectum, gluteus maximus, subcutaneous fat, and the interface between gluteus maximus and subcutaneous fat all showed advantages in cDWI_b1,000_ images (p <0.001).

### Interobserver Variability Evaluation of Objective Image Quality

The objective image quality parameters of both methods had excellent agreement (P <0.001 for each parameter) with ICC values ranging from 0.817 to 0.952 (**Table 5**).

**Table 5:** Interobserver Variability of Image Quality of DWI_b1,000_ and aDWI_b1,000_ images.

Parameters	cDWI_b1,000_ images	aDWI_b1,000_ images
SNR	0.944 (0.919, 0.962)	0.952 (0.930, 0.967)
CNR (mesorectum)	0.931 (0.900, 0.953)	0.817 (0.741, 0.872)
CNR (gluteus)	0.938 (0.910, 0.958)	0.868 (0.811, 0.908)
CNR (fat)	0.941 (0.914, 0.960)	0.870 (0.812, 0.910)
CNR (interface)	0.938 (0.907, 0.958)	0.858 (0.792, 0.904)
SIR (mesorectum)	0.941 (0.914, 0.960)	0.887 (0. 838, 0.922)
SIR (gluteus)	0.923 (0.888, 0.947)	0.930 (0.898, 0.952)
SIR (fat)	0.891 (0.842, 0.925)	0.910 (0.869, 0.938)
SIR (interface)	0.926 (0.890, 0.951)	0.849 (0.780, 0.898)

Data in parentheses are 95% confidence intervals.

aDWI_b1,000_, acquired diffusion-weighted imaging with b-value of 0 and 1,000 s/mm^2^; cDWI_b1,000_, computed diffusion images with b-value of 1,000 s/mm^2^ from 0 and 700 s/mm^2^.

## Discussion

Our results showed that the computed diffusion images with b-value of 1,000 s/mm^2^ had both higher subjective and objective image quality than that of the acquired DWI images. Additionally, despite being verified to have no significant difference between both diffusion images in the N and T4 staging performance, notable T staging and discrimination between T1–2 and T3–4 rectal cancers advantages were found when using T2W and the cDWI_b1,000_ images than T2W and aDWI_b1,000_ images. Therefore, cDWI_b1,000_ images generated from lower b-values may potentially serve as substitute for the acquired DWI with better image quality and equal or even better diagnostic performance in preoperative rectal cancer staging.

In recent years, increasing evidence shows that DWI provides added great benefit to conventional morphological sequences, namely, preoperative staging, the assessment of treatment response, and prediction of the status of lymph nodes (4, 26, 28, 31, 32). The routine use of DWI for rectal cancer imaging has recently been recommended in the expert consensus guidelines of the European Society of Gastrointestinal Abdominal Radiology (4). Image contrast on DWI varies greatly with the b-value. At higher b-values, tissues with high water molecule path lengths tend to lose signal rapidly, while image noise does not (5). However, images with high b-values are of great significance in tumor detection and staging/restaging for rectal cancer (9, 10, 13). Despite the clinical necessity of DWI with satisfying higher b-values, obtaining such images is needed for prolonged acquisition times. Computed DWI is a mathematical computation method that generates diffusion images of any b-value by using acquired DWI data with at least two different b-values. Based on the ADC value and a mono-exponential model, estimated ADC value was figured out and thus the computed diffusion images. From previous studies on phantom and different tumors, computed diffusion images can suppress background noise, maintain lesion signals, and lead to a better contrast between the cancerous lesions and normal tissue (9, 17, 18). In this study, we have computed diffusion images with b-value of 1,000 s/mm^2^ from lower b-values (0 and 700 s/mm^2^) and compared it with the conventionally acquired DWI images in terms of the image quality and diagnostic performance.

In our study, the cDWI_b1,000_ images demonstrated both significantly better subjective and objective image quality with greater background signal suppression, less distortion, and fewer artifacts than aDWI_b1,000_ images. However, DelPriore et al. found CNR was adequate but slightly higher for acquired versus computed images at the same b-value when employed in breast cancers (33). In their study, they employed diffusion images with 100 and 800 s/mm^2^ from phantom and 30 women patients to computed high b-value of 1,500 s/mm^2^. Different cancers, population and lower b-values used might be the reason for the inconsistency. In another article performed by Fukukura et al., the authors reported that computed diffusion with b-values of 1,500 and 2,000 s/mm^2^ generated from two b-values of 0 and 1,000 s/mm^2^ produced a significantly lower image quality than directly acquired images (16). It might be caused that the 1,000 s/mm^2^ they used as “lower b-value” suffered from lower SNR itself. Other previous studies on prostate cancer employed 3 lower b-values (<800 s/mm^2^) to computed diffusion images with b-value of 2,000 or 2,500 s/mm^2^ (18, 34). They found similar results as ours that computed diffusion images had higher image quality than acquired images. The significant improvement of image quality may relate to the higher SNR and less distortion artifact of the original images with lower b-value (<800 s/mm^2^) as its principle indicated.

When it comes to staging performance, overall accuracy was increased in each T stage with the use of the computed diffusion images. Moreover, the computed images exhibited better qualities in distinguishing T1–2 from T3–4 tumors. This improvement is meaningful for clinical decision-making, taking into consideration neoadjuvant therapy in patients with the latter, known as locally advanced rectal cancer. Overstaging may lead to overtreatment for patients with T1–2 tumors and an elevated risk for therapy-related morbidity and mortality, while the underestimation might lead to lower possibility of local control (35). In the present study, the computed diffusion images avoided 7 cases with T3 tumors from overstaging, and 5 cases with T3 tumors from underestimation, providing higher accuracy, sensitivity, and the specificity in detecting T3 tumors. It might be attributed to the higher confidence of computed diffusion images for identifying diffusion restriction because of efficient long T2 suppression. Unfortunately, there is no other similar research on rectal cancer to be compared with. Further study with a larger population is needed to confirm our results.

Speaking of N staging, we adopted the combination morphology-size-diffusion criteria using cDWI_b1,000_ and aDWI_b1,000_ diffusion images. No significant difference in each N stages were found using the cDWI_b1,000_ images compared to aDWI_b1,000_ images except for higher sensitivity of the cDWI_b1,000_ images in determining N1 tumors. Additionally, the cDWI_b1,000_ and aDWI_b1,000_ images showed similar moderate sensitivity and high specificity in identifying patients without metastatic nodes. Our results may approve the conclusions of Fornell-Perez et al. (29). They reported adding diffusion criteria did not demonstrate a clear advantage over the mixed morphology-size criteria.

There are some limitations in this study. First, the grouping criteria we used in the T stages could be roughly simple because we did not consider different prognoses of various T3 subgroups on the basis of the distance invasion outside the border of the muscularis propria (36). Large pT3 tumors that behave more like pT4 tumors are associated with poorer prognosis, whereas borderline pT3 tumors are known to have better prognosis as pT2 tumors do. Second, we only conducted the feasibility of computed high b-value of 1,000 s/mm^2^. Hausmann et al. reported ultra-high b-value of 2,000 s/mm^2^ may be beneficial in assessing both the primary and the residual tumor after CRT in rectal cancer (22). On the basis of our study, the value of computed ultra-high b-values from lower b-values would be explored in the future. Finally, the number of the whole study population and some subgroups including patients with T4 or N2 tumors in this study was relatively small. It might be attributed to that patients with locally advanced rectal cancer usually underwent adjuvant chemoradiation and then were excluded from the study.

In conclusion, the computed diffusion images with value of 1,000 s/mm^2^ provided significantly better image quality, lesion conspicuity, and diagnostic performance than the acquired DWI images with the same b-value. Therefore, the use of computed diffusion images from lower b-values might be a promising tool to be effective and feasible in routine work, helpful for better decision-making in clinical practice.

## Data Availability Statement

The original contributions presented in the study are included in the article. Further inquiries can be directed to the corresponding authors.

## Ethics Statement

The studies involving human participants were reviewed and approved by the Ethics Committee of the Ruijin Hospital. The ethics committee waived the requirement of written informed consent for participation.

## Author Contributions

YX and LW, validation, data curation and original draft preparation; JT, methodology; MF, investigation; CF, scanning sequences’ optimization; ZP, supervision; ZW and LZ, formal analysis and funding acquisition; FY, visualization; GC, QM, and HS, conceptualization, resources, project administration, review and editing. All authors contributed to the article and approved the submitted version.

## Funding

This work was supported by fund of the Shanghai Sailing Program (grant number 20YF1427200); the National Natural Science Foundation of China (grant number 81771789 and 62173223).

## Conflict of Interest

The author (CF) is an employee of Siemens Healthcare but did not have control over the data.

The remaining authors declare that the research was conducted in the absence of any commercial or financial relationships that could be construed as a potential conflict of interest.

## Publisher’s Note

All claims expressed in this article are solely those of the authors and do not necessarily represent those of their affiliated organizations, or those of the publisher, the editors and the reviewers. Any product that may be evaluated in this article, or claim that may be made by its manufacturer, is not guaranteed or endorsed by the publisher.
